# Kidney–placenta crosstalk in health and disease

**DOI:** 10.1093/ckj/sfac060

**Published:** 2022-04-15

**Authors:** Omar Cabarcas-Barbosa, Olivia Capalbo, Alberta Ferrero-Fernández, Carlos G Musso

**Affiliations:** Nephrology Department, Universidad Simon Bolivar, Barranquilla, Colombia; Physiology Department, Instituto Universitario del Hospital Italiano de Buenos Aires, Argentina; Physiology Department, Instituto Universitario del Hospital Italiano de Buenos Aires, Argentina; Physiology Department, Instituto Universitario del Hospital Italiano de Buenos Aires, Argentina; Facultad de Ciencias de la Salud, Universidad Simon Bolivar, Barranquilla, Colombia

**Keywords:** crosstalk, eclampsia, HELLP syndrome, kidney, placenta

## Abstract

Organ crosstalk allows the interaction between systems to adapt to a constant changing environment, maintaining homeostasis. The process of placentation and the new hormonal environment during pregnancy trigger physiological changes that modulate kidney function to control extracellular volume, acid–base balance and filtration of metabolic waste products. The bidirectional communication means that acute or chronic dysfunction of one organ can compromise the other. Abnormal placentation in pregnancy-related hypertensive disorders such as pre-eclampsia and HELLP (haemolysis, elevated liver enzymes and low platelet count) syndrome leads to the release of antiangiogenic factors that may cause kidney injury (thrombotic microangiopathy, glomeruloendotheliosis, mesangiolysis and vasoconstriction of peritubular vessels). These hypertensive disorders are a key cause of kidney injury in gestation, which increases maternal morbimortality and adverse foetal outcomes. Conversely, prior kidney injury or causes of kidney injury (diabetes, lupus, glomerulonephritis or other forms of chronic kidney disease) increase the risk of developing hypertensive pregnancy disorders, providing a baseline higher risk. Inherited kidney diseases are a special concern, given the potential for genetic predisposition to kidney disease in the foetus. Understanding the bidirectional potential for compromise from placenta to kidney and vice versa provides a better framework to limit damage to both organs and improve maternal and foetal outcomes.

## INTRODUCTION

Neuron and bloodstream signalling via hormones, transmitters, chemical mediators and paracrine interactions within the same tissue provide communication pathways within and between organs, allowing the orchestration of body functions to maintain homeostasis. This crosstalk allows coordination and adaptation to a constantly changing environment, but its bidirectional or multidirectional nature also enables the extension of injury from one organ to others and exchange of inflammatory mediators.

The process of placentation and the new hormonal milieu during pregnancy trigger a series of physiological changes. The kidney plays a key role in maintaining and managing the plasma volume expansion necessary for optimal maternal and foetal perfusion, as well as controling adequate blood pressure. Hence a diseased kidney will undergo poor adaptation and provide an adverse scenario that increases the risks of complications of pregnancy and delivery. In contrast, gestational disease will also impact on the kidney, which will in turn malfunction, feeding the loop of damage.

**FIGURE 1:**
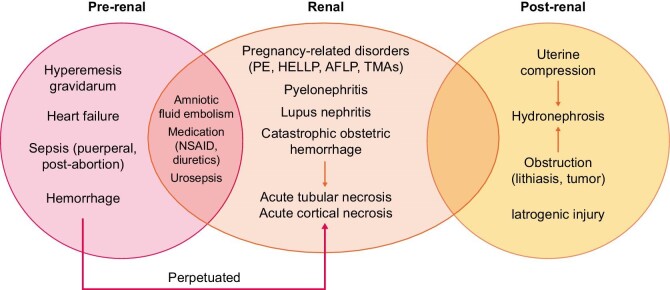
Pre-renal, renal and post-renal causes of AKI during pregnancy. NSAID, non-steroidal anti-inflammatory drug; PE, pre-eclampsia; AFLP, acute fatty liver of pregnancy.

**FIGURE 2:**
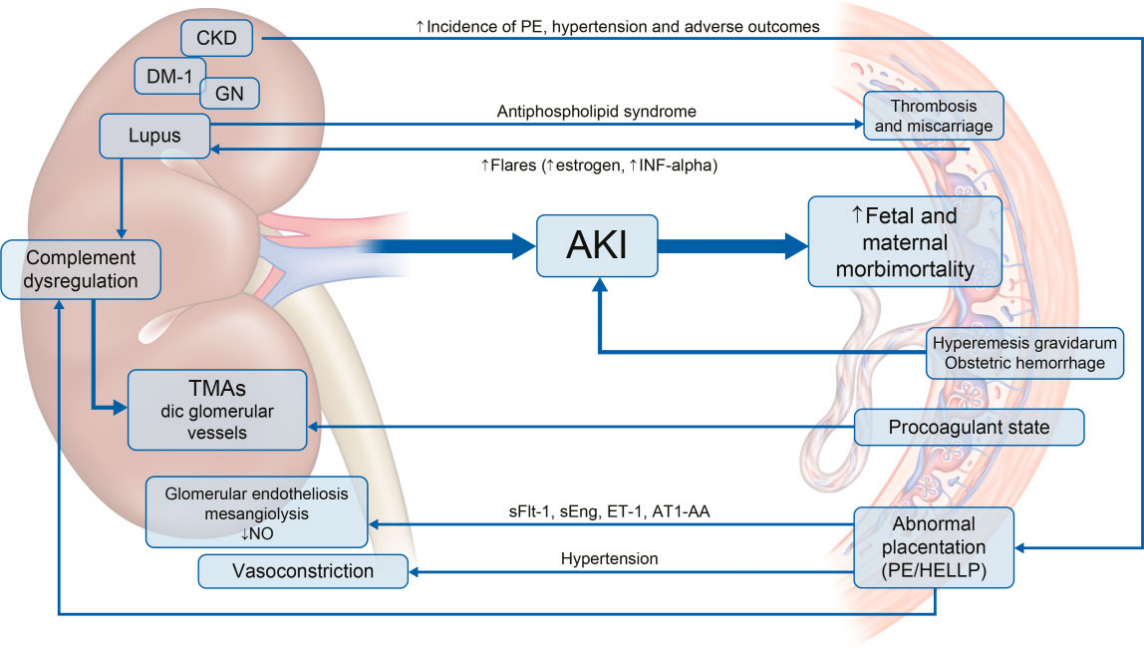
Kidney–placenta crosstalk. PE, pre-eclampsia; DM-1, diabetes mellitus type 1; DIC, disseminated intravascular coagulation; ET-1, endothelin 1; AT1-AA, agonistic angiotensin receptor 1 autoantibodies.

Pre-eclampsia, acute fatty liver of pregnancy, HELLP (haemolysis, elevated liver enzymes and low platelet count) syndrome and thrombotic microangiopathies (TMAs) are considered pregnancy-related disorders. They are associated with increased maternal morbimortality and adverse foetal outcomes such as lower mean gestational age at delivery, lower birthweight and increased perinatal mortality. The risk of adverse outcomes is further increased if acute kidney injury (AKI) develops in these conditions and pregnancy-related disorders are the most common cause of AKI in pregnancy. In India, pre-eclampsia accounted for 47% of pregnancy AKI, HELLP syndrome for 6.8% and acute fatty liver of pregnancy for 3.8% [1]. In Canada, the increased risk in pregnancy AKI was restricted to women with hypertensive disorders, especially those with pre-eclampsia [2]. A prospective study in Malawi and a retrospective study in Tunisia reported that pre-eclampsia accounted for 73% and 66% of AKI cases, respectively [3, 4]. In a systematic review and meta-analysis of 11 studies, women with pregnancy AKI had a greater probability of caesarean section, haemorrhage, placenta abruption, maternal death, longer intensive care unit stays and stillbirth [5].

Women of childbearing age with chronic kidney disease (CKD) have an increased risk of adverse pregnancy-related outcomes, including increased perinatal death and prematurity, which further increases with worsening CKD [6]. CKD also increases the risk of developing pre-eclampsia and other pregnancy-related disorders that may aggravate the kidney condition.

The purpose of this review article is to explore pathological mechanisms of pregnancy-related diseases that adversely impact on the kidney and pathological mechanisms of kidney disease that adversely impact on pregnancy-related diseases in order to optimize care during gestation and decrease the risk of pregnancy-related disorders, AKI and associated adverse outcomes.

### Placenta–kidney perspective

From the placenta–kidney perspective, we should understand the physiological kidney changes during pregnancy, the pathogenesis of pregnancy-related diseases and the impact on healthy kidneys and pregnancy effects on kidney disease.

### Physiological changes of the kidney during pregnancy

Hormonal and anatomic changes during pregnancy result in an ∼40–50% increase in glomerular filtration rate (GFR), which peaks during the first trimester and results in increased urinary protein excretion [7–9]. The increase in GFR is driven by increased cardiac output, increased renal blood flow and decreased blood pressure due to vasodilation and plasma volume expansion resulting from several signalling molecules that decrease kidney excretion of sodium.

Vasodilation in the first stages of gestation is mainly related to relaxin, a hormone produced by the decidua, placenta and corpus luteum that activates the RXFP1 receptor to exert both rapid (minutes) and sustained (hours to days) vasodilatory actions through different molecular mechanisms that have increased endothelial nitric oxide (NO) availability as a final common pathway [10–12]. Thus it leads to generalized renal vasodilation, decreased renal afferent and efferent arteriolar resistance and a subsequent increase in renal blood flow and GFR. Relaxin administration to male and female non-pregnant rats reproduced the haemodynamic changes observed in pregnancy [11].

The expansion of plasma volume is due to increased salt and water retention, with a net excess of water, resulting in decreased osmolality. There is an interaction between natriuretic and anti-natriuretic factors. On the one hand, the renin–angiotensin–aldosterone system (RAAS) is upregulated and oestrogens increase angiotensinogen synthesis by the liver. Aldosterone and deoxycorticosterone from progesterone conversion promote sodium retention. On the other hand, progesterone competes with tubular mineralocorticoid receptor agonists to limit sodium retention. The increase in natriuretic peptides and the higher GFR promote salt excretion [11]. A lower threshold of osmoreceptors for antidiuretic hormone release favours water gain that reduces the oncotic pressure, intensifying the increase in GFR.

The physiological hydronephrosis in pregnancy is mainly explained by anatomic compressive factors. The prevalence of right-kidney hydronephrosis supports this, as the right ureter crosses the pelvis and iliac vessels in a more acute angle than the left, which descends parallel to the vessels. Although it is a normal state, it is worth noting that urine stasis can increase the risk of pyelonephritis, thus the importance of detecting and treating asymptomatic bacteriuria [10]. Progesterone also reduces the ureteral tone and may contribute to hydronephrosis.

### Pathogenesis of pregnancy-related diseases and impact on healthy kidneys

Pre-eclampsia is defined by new-onset hypertension (systolic blood pressure ≥140 mmHg or diastolic blood pressure ≥90 mmHg on two occasions 4 h apart or systolic blood pressure ≥160 mmHg or diastolic blood pressure ≥110 mmHg confirmed within minutes) and new onset of either proteinuria ≥300 mg in 24-h urine or a protein:creatinine ratio ≥300 mg/g or thrombocytopenia (<100 000 × ${10^9}$/L) or increased serum creatinine (>1.1 mg/dL or doubling of serum creatinine in the absence of other kidney disease), or increased liver transaminases (≥2-fold over the upper limit of the normal concentrations) or pulmonary oedema or new-onset and persistent headache not accounted for by alternative diagnoses and not responding to usual doses of analgesics or visual symptoms (e.g. blurred vision, flashing lights or sparks, scotomata) [13].

The placenta and, specifically, impaired placentation play a key role in the pathogenesis of pre-eclampsia. A two-stage model was proposed in which deficient uterine spiral artery remodelling leads to placental ischaemia (stage 1) and the consequent release of antiangiogenic factors, such as soluble fms-like tyrosine kinase 1 (sFlt-1) or soluble vascular endothelial growth factor receptor-1 (sVEGFR-1) and soluble endoglin (sEng), leads to generalized endothelial dysfunction (stage 2). Defective trophoblast invasion of the spiral arteries may be favoured by defective cytotrophoblast differentiation from an epithelial phenotype to an endothelial one that prevents the development of lower resistance vessels accommodating the increased blood flow that nourishes the foetus [14]. Immunological and environmental factors together with genetic predisposition may also contribute [15]. Diverse immune abnormalities have been described from agonistic antibodies to the angiotensin AT-1 receptor to immunologic abnormalities, similar to those observed in organ rejection [16]. The heme oxygenase enzyme is usually upregulated in hypoxia, as it generates the vasodilatory product carbon monoxide. However, heme oxygenase 1 (HO-1) gene expression was decreased in chorionic villous samples from 11-weeks pregnant women who would develop pre-eclampsia [16]. Among antiangiogenic factors released by the hypoxic placenta, sFlt-1 is a decoy receptor that prevents VEGF binding to receptors on podocytes and renal endothelial cells. The resultant endothelial dysfunction causes cell ballooning with consequent capillary obstruction (glomerular endotheliosis), mesangiolysis, reduced NO and prostacyclin production and glomerular filtration barrier dysfunction. The normal paracrine signalling of VEGF released from podocytes is also impaired [17]. Eng is a coreceptor for transforming growth factor (TGF)-β and sEng is an antiangiogenic factor that inhibits TGF-β1 activation of endothelial nitric oxide synthase in endothelial cells, further reducing NO levels [18]. Endothelial dysfunction results in the release of thromboxane A2, complement activation and an imbalance of anti- and procoagulant regulators and leads to disseminated coagulation in glomerular vessels that decreases GFR [19]. Angiotensin receptor 1 agonistic autoantibodies increase oxidative stress and the secretion of endothelin 1 (ET1), a potent vasoconstrictor, contributing to hypertension [20]. Placental hypoxia promotes placental cell death, resulting in the release of cell-free DNA and other danger-associated molecular patterns into the maternal circulation that may interact with increased expression of receptors such as toll-like receptor 4 to exacerbate inflammatory responses [21].

HELLP syndrome is considered a severe form of pre-eclampsia, as it has a higher maternal morbimortality. It is defined as elevated lactate dehydrogenase (>600 IU/L), aspartate and alanine aminotransferases twice the normal upper limit and thrombocytopenia (<100 000 × ${10^9}$/L) [13]. The abnormal placentation pathway is also responsible, leading to endothelial dysfunction and the release of antiangiogenic factors. Elevated titres of angiotensin receptor 1 agonistic autoantibodies and ET1 in plasma were registered in HELLP patients and administration of angiotensin receptor 1 agonistic autoantibodies in rats elicited typical HELLP features [19]. Activation of the alternative complement pathway was also evidenced [22]. Around 50% of women with HELLP syndrome developed AKI and AKI was associated with higher maternal mortality [23].

TMAs involve endothelial dysfunction leading to platelet aggregation in small vessels that cause mechanical haemolytic anaemia, thrombocytopenia and tissue ischaemia, with predominant kidney and neurological disease. Thrombotic thrombocytopenic purpura (TTP) and atypical haemolytic uraemic syndrome (aHUS) may be observed in pregnancy. Gestation itself is a procoagulant state and a trigger for complement activation (as for pre-eclampsia and HELLP, which may be considered a form of TMA) that causes endothelial cell injury. TTP may be triggered by pregnancy in persons with congenital ADAMTS-13 (a disintegrin and metalloproteinase with a thrombospondin type 1 motif, member 13) deficiency or acquired anti-ADAMTS-13 immunoglobulin G antibodies [22]. aHUS may be triggered by pregnancy in persons with complement dysregulation resulting from genetic (mutations in genes encoding for factor H, factor I, C3) or acquired causes [24]. aHUS may be triggered after delivery, when inflammation, haemorrhage and circulating foetal cells activate the alternative complement pathway [17].

Acute fatty liver of pregnancy shares features with pre-eclampsia with severe features or HELLP syndrome. It may be caused by a foetal deficiency of mitochondrial beta-oxidation of fatty acids that leads to accumulation of free fatty acids and intermediate products of metabolism that cross the placenta and cause fatty deposits in liver and kidney tubular epithelium. It is characterized by increased liver enzymes, hyperbilirubinaemia, coagulopathy (disseminated intravascular coagulation and thrombocytopenia), leucocytosis and release of free radicals, all of which contribute to the development of AKI [25, 26].

Overall, these entities can cause AKI through different mechanisms, ranging from direct effects on the kidney (TMA, glomerular endotheliosis, mesangiolysis and vasoconstriction) to pre-renal AKI due to haemorrhage, hypovolaemia or oedema, leading to acute tubular necrosis and/or acute renal cortical necrosis [27] that increases perinatal mortality by 3-fold [24] and may lead to CKD [28].

### Pregnancy effects on kidney disease

Gestation can increase the severity of underlying CKD. The pathogenesis may be disease specific. For example, systemic lupus erythematous (SLE) is an autoimmune disease more frequent in women of childbearing age. Patients with active SLE and lupus nephritis have an increased risk of adverse pregnancy outcomes [24]. Around 3–5% of women with SLE develop severe flares after pregnancy [7]. Suggested mechanisms include high oestrogen levels that interact with regulatory T cells and an upregulation of placenta-expressed interferon-α [17].

The incidence of preterm delivery and development of proteinuria and hypertension was higher in women with glomerulonephritis (GN) than in controls. Women with immunoglobulin A nephropathy were at higher risk for development of pre-eclampsia [17, 29]. Women with renal-relevant pregnancy complications had a shorter latency time to a diagnosis of GN than those with uncomplicated pregnancy [30]. However, it is unclear whether pre-existent undiagnosed GN may have favoured pregnancy complications, GN was misdiagnosed as pre-eclampsia or there are further pathogenic links between them.

### Kidney–placenta perspective

From the kidney–placenta perspective, we should understand the impact of both AKI and CKD on pregnancy.

### AKI effects on pregnancy

Pregnant women may develop AKI from multiple causes beyond the pregnancy-related disorders mentioned above. With the premise of organ crosstalk in mind, both AKI arising from abnormal placentation and AKI from unrelated conditions are associated with adverse outcomes and increased perinatal and maternal morbimortality. In the past, AKI was mainly related to complications from septic abortions and post-partum haemorrhages. Pre-eclampsia is now a leading cause of AKI mainly in developed countries, while in developing countries where abortion is illegal, septic abortion is a frequent cause of AKI [17, 28, 31]. Pre-renal AKI can also result from heart failure, hyperemesis gravidarum and haemorrhage, while sepsis may complicate pyelonephritis [25]. Drugs such as diuretics and non-steroidal anti-inflammatory drugs that can cause pre-renal AKI are not generally used during pregnancy. Additionally, the most prevalent causes change according to the gestational period. In the first trimester, these are septic abortions and hyperemesis gravidarum and for the second and third trimesters, hypertensive disorders of pregnancy, acute liver disease, TMAs and autoimmune diseases [24, 25]. The most characteristic histological lesion in hypertensive disorders of pregnancy associated with AKI is glomeruloendotheliosis. However, tubular injury also occurs.

Distant organ failure in the context of AKI is well studied and provides the basis for the increased mortality in patients with severe AKI. Kidney failure may be complicated by cardiorenal syndrome, hepatorenal syndrome, acute lung injury and even brain injury to which AKI-associated metabolic derangements and inflammation may contribute [32]. Finally, complications can potentially be generated from interventions such as renal support therapy and infections associated with an intravascular device, among others. Perfusion of the foetoplacental circulation can be affected by the haemodynamic changes associated with kidney replacement therapy [32].

Additionally, systematic reviews have reported that pregnant women with AKI have a higher risk of post-partum haemorrhage {odds ratio (OR) 1.2 [confidence interval (CI) 1.02–1.56]}, HELLP syndrome [OR 1.86 (CI 1.4–2.46)], placental abruption [OR 3.13 (CI 1.96–5.02)], perinatal death [OR 3.39 (CI 2.76–4.18)] and low birthweight [5]. Hypertensive disorders during pregnancy by themselves can be associated with a risk of preterm delivery and consequently a decrease in nephron mass, making this a risk factor for developing CKD in adulthood [33–35].

Although current evidence is limited, some studies suggest that episodes of AKI are associated with an increased risk of CKD and the development of pre-eclampsia in the mother [36].

### CKD effects on pregnancy

The prevalence of CKD increases with age. Thus, the overall prevalence of CKD among pregnant women is low. However, CKD increases the risk of adverse maternal and foetal outcomes in proportion to the stage and severity of the disease. Among pregnant women with CKD, 40% developed pre-eclampsia, 48% developed anaemia and 56% developed chronic hypertension [37]. Additionally, moderate and severe CKD cause increased foetal growth restriction and preterm deliveries [38, 39].

The cause of CKD may also be associated with pregnancy complications, even when kidney function is preserved. Women with type 1 diabetes are at increased risk of developing pre-eclampsia, independent from baseline proteinuria. This appears to be dependent on diabetic vascular dysfunction that increases the susceptibility to placental hypoperfusion [40]. A prospective follow-up of 83 pregnant patients with long-standing type 1 diabetes mellitus assessed whether vascular dysfunction in the early stages of pregnancy predicted the risk of developing pre-eclampsia. There were no significant differences in vascular dysfunction parameters, VCAM-1 and ICAM-1 serum levels between patients who developed pre-eclampsia and those who did not (*P* = 0.77) [40]. Diabetes mellitus has been associated with foetal growth impairment, stillbirths, preterm delivery and malformations, and this risk is increased in the presence of diabetic nephropathy [6].

In the pregnant population with lupus, the most frequent maternal complications include relapse of lupus (25.6%), hypertension (16.3%), lupus nephritis (16.1%) and pre-eclampsia (7.6%). Patients with established lupus nephritis had a higher risk of hypertension (*P* < 0.001) and premature birth (*P* = 0.020). Anti-phospholipid antibodies were associated with arterial hypertension (*P* = 0.029), premature delivery (*P* = 0.004) and abortion (*P* = 0.016) [40].

Finally, a frequently overlooked fact is the overrepresentation of inherited kidney disease as a cause of CKD in pregnant women as compared with the wider CKD population [41]. Thus inherited kidney disease accounted for ∼15% of prevalent <44-year-old persons with CKD in the 2016 Registro Madrileño de Enfermedades Renales [42]. Additionally, inherited kidney diseases are likely underdiagnosed [43]. This may be especially true for some autosomal dominant conditions such as Alport syndrome, MYH9-associated disease or autosomal dominant tubulointerstitial kidney disease [44–47]. In these cases, any adverse impact on placentation or the foetus may occur on a background of foetal genetic predisposition to kidney disease.

## CONCLUSION

Placenta and kidneys are two metabolically active organs that during pregnancy have a complex relationship with the aim of maintaining homeostasis, and given this bidirectional nature, acute or chronic dysfunction of one can lead to compromise of the other. The process of placentation and hormonal release during pregnancy leads to multiple physiological changes in the kidney that induce volume retention and modulate acid–base balance and the excretion of metabolic waste products. An imbalance between antiangiogenic and angiogenic factors in placentation disorders such as pre-eclampsia and HELLP syndrome results in hypertension that may cause kidney injury. In contrast, prior kidney injury or even causes of kidney injury may increase the risk of placentation disorders and adverse maternal and foetal outcomes. Understanding the bidirectional potential for compromise from placenta to kidney and vice versa provides a better framework to limit damage to both organs and improve maternal and foetal outcomes.
